# Predicting the true extent of glioblastoma based on probabilistic tractography

**DOI:** 10.3389/fnins.2022.886465

**Published:** 2022-09-21

**Authors:** David Kis, Laszlo Szivos, Mark Rekecki, Bayan Salam Shukir, Adrienn Mate, Katalin Hideghety, Pal Barzo

**Affiliations:** ^1^Department of Neurosurgery, Faculty of Medicine, University of Szeged, Szeged, Hungary; ^2^Department of Oncology, Faculty of Medicine, University of Szeged, Szeged, Hungary

**Keywords:** probabilistic tractography, glioblastoma, tumor recurrence, extended survival, infiltration

## Abstract

Glioblastoma is the most frequent type of primary brain tumors. Despite the advanced therapy, most of the patients die within 2 years after the diagnosis. The tumor has a typical appearance on MRI: a central hypointensity surrounded by an inhomogeneous, ring-shaped contrast enhancement along its border. Too small to be recognized by MRI, detached individual tumor cells migrate along white matter fiber tracts several centimeters away from the edge of the tumor. Usually these cells are the source of tumor recurrence. If the infiltrated brain areas could be identified, longer survival time could be achieved through supratotal resection and individually planned radiation therapy. Probabilistic tractography is an advanced imaging method that can potentially be used to identify infiltrated pathways, thus the real extent of the glioblastoma. Our study consisted of twenty high grade glioma patients. Probabilistic tractography was started from the tumor. The location of tumor recurrence on follow-up MRI was considered as the primary infiltrated white matter tracts. The results of probabilistic tractography were evaluated at thirteen different thresholds. The overlap with the tumor recurrence of each threshold level was then defined to calculate the sensitivity and specificity. In the group level, sensitivity (81%) and specificity (90%) were the most reliable at 5% threshold level. There were two outliers in the study group, both with high specificity and very low sensitivity. According to our results, probabilistic tractography can help to define the true extent of the glioblastoma at the time of diagnosis with high sensitivity and specificity. Individually planned surgery and irradiation could provide a better chance of survival in these patients.

## Introduction

Glioblastoma is the most frequent type of primary brain tumors. The majority of patients die within 16–20 months after the diagnosis despite all the advanced therapy (Yan et al., 2009). Currently, the gold standard therapy for glioblastoma consists of maximum safe resection (more than 90% of the tumor mass should be removed) that is followed by the Stupp protocol (Stupp et al., 2005; Louis et al., 2021; Ostrom et al., 2021).

Glioblastoma is a rapidly growing, infiltrative malignant tumor originating from the glial cells of the white matter. Tumor cells tend to propagate along white matter fiber tracts. In an advanced stage, the tumor can infiltrate gray matter as well (Wirsching et al., 2016). Histologically, glioblastoma is characterized by atypical cells, a high mitotic rate, glomeruloid vascular proliferation, and necrosis (Demuth and Berens, 2004).

Currently, the most sensitive *in vivo* diagnostic modality is contrast-enhanced T1 MRI. The appearance of the tumor on MRI images is very typical. Irregular ring-shaped contrast enhancement can be seen with a hypointense necrotic center, usually with prominent peritumoral edema and mass effect (Young, 2007; Shukla et al., 2017). Previous studies have shown that the tumor mass identifiable on the MRI images does not correspond to the true extent of the tumor (Demuth and Berens, 2004). Due to the tumor’s infiltrative growth, glioblastoma cells can be present at a distance of 5 to 10 cm from the contrast-enhancing border. Some studies have demonstrated that glioblastoma cells can be present anywhere in the brain, including the contralateral hemisphere (Tonn and Goldbrunner, 2003).

Tumor cells are migrating from the tumor mass along fiber tracts, invading brain regions far away from the original site of the tumor. Although the tumor mass and infiltrative part form one structural unit, only the tumor mass can be visualized easily on standard MRI images (Petrecca et al., 2013; Lemee et al., 2015). The infiltrative component can be considered as an abnormal “fiber tract” that first invades and then destroys normal brain (Virga et al., 2019).

The recurrence of glioblastoma usually originates from these infiltrative tumor cells, which is supported by the observation that tumor progression often appears in the white matter surrounding the resection cavity (Milano et al., 2010; Petrecca et al., 2013).

This aggressive, infiltrative feature of the tumor makes the available treatment options less effective. The surgical resection is aimed at removing the tumor mass visible on the MRI images. The remaining tumor cells, which may be present in significant amounts even if the post-operative MRI shows a “tumor-free” status, are treated with irradiation and chemotherapy (Stupp et al., 2005). After a gross total or subtotal resection, the patients’ survival depends on how the remaining tumor cells respond to oncological treatment. These treatments are more effective if the residual tumor volume is lower (Roelz et al., 2016).

As opposed to chemotherapy, which is a systematic treatment, surgery and irradiation are focal treatments. In the case of glioblastoma, these modalities are individually tailored to achieve the best results. Surgery is considered successful if no contrast enhancement is visible on the post-operative MRI images or if the residual tumor is less than 10% of its original size. Radiation therapy typically has a standard planning protocol. Irradiation is focused to an area that exceeds the border of the resection cavity/the residual tumor margins by 1 to 3 cm to destroy the remaining infiltrative glioblastoma cells (Barani and Larson, 2015).

There are several factors that define the patients’ survival, such as age, comorbidities, extent of resection, location of tumor, Karnofsky Performance Scale and molecular markers (e.g., MGMT promoter methylation, IDH mutant or wild type) (Lacroix et al., 2001; Bauchet et al., 2010). Recent studies have shown that gross total resection is an independent prognostic factor associated with improved clinical outcome (Wykes et al., 2021). Theoretically, better therapeutic effects and longer survival would be possible if the residual tumor volume could be minimized. It can be accomplished through supratotal tumor resection (when the resection margins exceed the contrast-enhancing border) or individually planned irradiation that focuses the beam to brain regions actually infiltrated by the tumor (De Bonis et al., 2013). The problem is that standard MRI sequences cannot identify infiltrated brain regions, that is, cannot determine the true extent of the tumor (Shukla et al., 2017; de Leeuw and Vogelbaum, 2019). FLAIR and MR Spectroscopy (MRS) are two MRI modalities which were introduced in the last decade to identify the infiltrative part of the glioblastoma. Although both sequences have promising results, their routine application is limited. Using FLAIR to guide surgical resection and irradiation has not resulted in longer survival (Garrett et al., 2017; Altieri et al., 2019). MRS has a low resolution which makes it challenging to use for such precise image guided procedures (Deviers et al., 2014).

Tractography is an advanced application of diffusion MRI. Based on the diffusion movement pattern of the water molecules, white matter fiber tracts can be visualized (Le Bihan et al., 1992; Mascalchi et al., 2005). The diffusion pattern is characterized by anisotropy. A more directional diffusion results in higher anisotropy. Hence fiber tract reconstruction is more certain in those brain regions with high anisotropy. In the last decade tractography has an evolving role in preoperative mapping and image guided therapy of brain tumors (Costabile et al., 2019). There are two types of tractography algorithms, the deterministic and probabilistic methods (Yamada et al., 2009).

Conventional diffusion tensor imaging-based deterministic tractography can visualize the major white matter pathways reliably (Mori and van Zijl, 2002; Mukherjee et al., 2008). Therefore, it can be used to identify white matter fibers around the tumor (e.g., corticospinal tract) and help to plan the surgical trajectory to avoid injuring important pathways. The main limitation of deterministic tractography is that in regions with low anisotropy values, such as cortical and subcortical gray matter, branching pathways and crossing fibers, it cannot identify fibers reliably (Yamada et al., 2009).

The ball and sticks model-based probabilistic tractography has several advantages and overcomes the above limitation. It tracks fibers in regions with low anisotropy values and visualizes crossing fibers. Moreover, it provides quantitative measures representing the connectivity distribution of the seed region. It can be thresholded to exclude false positive results (Behrens et al., 2003b, 2007). Probabilistic tractography is a powerful tool to identify white matter fiber tracts with a high certainty. Potentially, it can be also used to identify white matter fiber tracts that are infiltrated by the tumor (Behrens et al., 2007; Kis et al., 2014).

In our study, we aimed to identify the true extent of glioblastoma using probabilistic tractography in a retrospective manner. Tumor recurrence was identified on follow-up MRI images, and its sites were considered the brain regions that had originally been infiltrated by the tumor. Probabilistic tractography was performed on the preoperative MRI images, and the results were compared with the location of tumor recurrence at different threshold levels.

## Materials and methods

### Patient population

Patients were included in the study retrospectively from those who had undergone surgical treatment at our department between 2010 and 2021. Inclusion criteria were the followings: 1, The diagnosis were primary glioblastoma or grade III anaplastic oligodendroglioma. 2, All patients underwent either subtotal or total tumor resection during the primary surgery. Based on post-operative MRI images (acquired within 48 h), subtotal resection was defined as the remaining contrast-enhancing tumor was less than 10% of its original volume, while greater extent of remaining tumor is considered partial resection. Total resection was achieved when no contrast enhancement was visible (Ahmed et al., 2019). The surgical resection was followed by the Stupp protocol (Stupp et al., 2005). 3, All patients had preoperative DTI scans in addition to the routine head MRI protocol. 4, Follow-up MRI scans were acquired every 3 months. A total of 96 adult (>18 years) patients were screened and 20 were enrolled to the study. All of them had preoperative DTI. The two main reason why patients were excluded are 1, lost to follow-up or 2, partial surgical resection.

The first follow-up MRI was used to identify infiltrated fiber tracts that had prominent tumor recurrence in the white matter surrounding the resection cavity. The study was approved by the institutional review board, and written informed consent was obtained from all patients.

Patients’ data are summarized in Table 1.

**TABLE 1:** Patients’ clinical data are summarized in this table.

	Age (years) and Sex	PFS (months)	OS (months)	Time of diagnosis	Date of recurrence	Date of last follow-up/Death	Side	Localization	Histology
1.	52 Male	5	26	2012. 09	2013. 02	2014. 11	Left	Temporalis	Glioblastoma
2.	53 Male	4	12	2011. 02	2011. 06	2012. 02	Left	Temporo-parietalis	Glioblastoma
3.	47 Male	4	20	2010. 09	2011. 01	2012. 05	Left	Frontalis	Glioblastoma
4.	68 Male	13	20	2013. 01	2014. 02	2014. 09	Left	Temporalis	Glioblastoma
5.	54 Female	34	35	2010. 04	2013. 02	2013. 03	Left	Frontalis	Glioblastoma
6.	67 Male	5	12	2012. 02	2012. 07	2013. 02	Left	Temporalis	Glioblastoma
7.	26 Male	28	46	2010. 03	2012. 08	2014. 01	Right	Frontalis	Glioblastoma
8.	29 Male	47	64	2012. 05	2016. 04	2017. 09	Left	Fronto-parietalis	Oligodendroglioma –Gr 3
9.	34 Male	6	11	2010. 12	2011. 05	2011. 10	Left	Frontalis	Glioblastoma
10.	67 Female	37	37	2012. 06	2015. 07	2015. 07	Right	Frontalis	Oligodendroglioma –Gr 3
11.	51 Female	61	72	2011. 02	2016. 03	2017. 02	Left	Parietalis	Oligodendroglioma –Gr 3
12.	39 Male	22	47	2010. 07	2012. 05	2014. 06	Right	Parieto-occipitalis	Glioblastoma
13.	55 Male	2	4	2021. 02	2021. 04	2021. 06	Right	Temporo-parieto-occipitalis	Glioblastoma
14.	77 Female	8	10	2019. 10	2020. 06	2020. 08	Left	Temporo-parietalis	Glioblastoma
15.	68 Male	8	10	2020. 04	2021. 12	2022. 02	Left	Frontalis	Oligodendroglioma –Gr 3
16.	59 Male	2	3	2021. 10	2021. 12	2022. 02	Left	Frontalis	Glioblastoma
17.	56 Male	2	28	2019. 11	2020. 01	2022. 03	Left	Parietalis	Glioblastoma
18.	46 Female	1	2	2021. 12	2022. 01	2022. 02	Left	Parietalis	Glioblastoma
19.	36 Male	3	6	2020. 10	2021. 01	2021. 04	Left	Parietalis	Glioblastoma
20.	46 Male	4	15	2019. 11	2020. 03	2021. 02	Right	Frontalis	Glioblastoma

PFS, progression free survival; OS, overall survival.

### Imaging

Preoperative and follow-up MRI images were used in this study. Scanning was performed using a 3-T GE Signa Excite scanner. Two sequences of preoperative MRI were used: (1) contrast-enhanced high-resolution 3D axial FSPGR-T1; and (2) DTI images. For the follow-up MRI, only the contrast-enhanced high-resolution 3D axial FSPGR-T1 sequence was used.

Contrast-enhanced high-resolution T1-weighted scan parameters: 3D FSPGR fast spoiled gradient echo: repetition time [TR]/echo time [TE], 10.3/4.2 ms; flip angle, 15°; ASSET 2; field of view [FOV], 25 cm × 25 cm; matrix, 256 × 256; slice thickness, 1 mm.

Diffusion-weighted image parameters: DTI: TR/TE, 11,500/98.4 ms; flip angle, 90°; FOV, 24 cm × 24 cm; matrix, 80 × 80; slice thickness, 3 mm; ASSET: 2; *b*-value = 1,000 s/mm^2^, in 25 independent directions and 1 non-gradient set (*b*-value = 0 s/mm^2^).

Scans covered the whole head. Total scan time was 18–27 min, including all sequences (FLAIR, non-contrast and contrast-enhanced T1, T2, DWI, DTI, SWI).

### Data pre-processing

According to the method previously described by Behrens et al., MRI data were processed using tools from the FMRIB Software Library (FSL, version 5.0.7; Oxford Centre for Functional MRI of the Brain (FMRIB), United Kingdom)^1^ (Behrens et al., 2003a). Compressed NifTI images were created from the original DICOM files using Chris Rordens’ MRICron software (Rorden et al., 2007). Diffusion images were visually checked for artifacts. Data quality was satisfactory in all subjects; therefore, no volume was discarded. The standard preprocessing steps were taken: eddy current and motion correction (affine registration of the diffusion volumes to the b0 volume), adaptation of the b-matrix (Leemans and Jones, 2009) by eddy tool (FSL 6.0.1) (Andersson and Sotiropoulos, 2016), skull stripping, reconstruction of diffusion tensors, and estimation of diffusion parameters (Smith, 2002). For each patient, DTI images and preoperative FSPGR–T1-weighted images were registered to each other (6 degrees of freedom, cost function: mutual information, interpolation: trilinear). For analyses in the standard space, affine registration (12 degrees of freedom, cost function: correlation ratio, interpolation: trilinear) of each patient’s preoperative and follow-up FSPGR-T1 to standard T1 images (Montreal Neurological Institute, MNI152 1 mm brain) was done. Transformation matrices were created in every registration step. Image registration was performed with FMRIB’s linear registration tool (FLIRT) (Jenkinson and Smith, 2001; Jenkinson et al., 2002).

### Defining masks

In this study, we used two types of masks: (1) tumor masks (primary tumor and tumor recurrence masks); and (2) cortical and subcortical white matter masks.

Tumor masks were used to identify the true extension of the glioblastoma. The primary tumor mask served as a seed mask for probabilistic tractography, and the tumor recurrence mask was considered to represent the primary infiltrated brain regions.

The cortical and subcortical white matter masks were used to identify brain regions infiltrated by the tumor and where the tumor recurred.

#### Cortical and subcortical white matter masks

**FIGURE 1:**
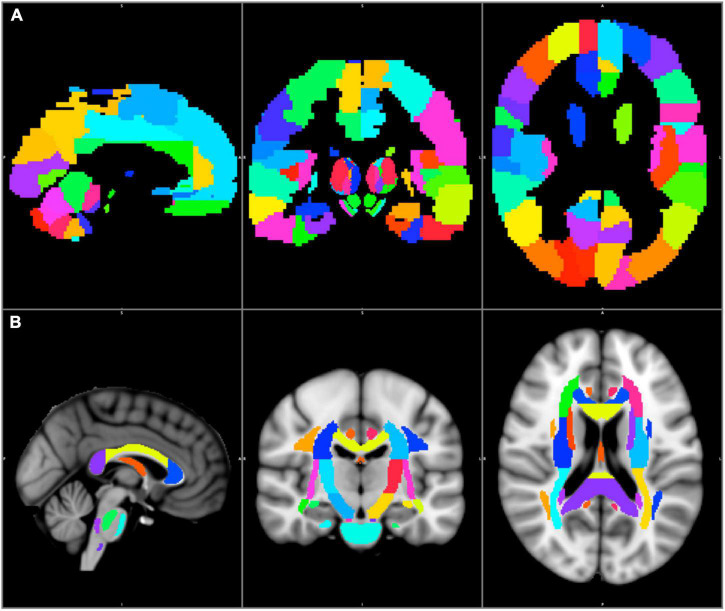
The AAL3 cortical **(A)** and the JHU–ICBM–DTI–81 white matter labels subcortical **(B)** regions are shown in the MNI152 1 mm space.

The standard brain was divided into distinct subcortical and cortical subregions. The standard JHU ICBM–DTI–white matter–81 labels map was used to segment the white matter into 54 subregions in the left and right hemispheres each, and 6 midline structures (Mori et al., 2008). The standard AAL3 map was used to segment the cortex into 84 subregions on either side (Rolls et al., 2020). The JHU–ICBM–DTI–white matter–81 labels and AAL3 maps were in the MNI152 1 mm space (Hua et al., 2008; Rolls et al., 2020). Figures 1A,B

#### Tumor masks

**FIGURE 2:**
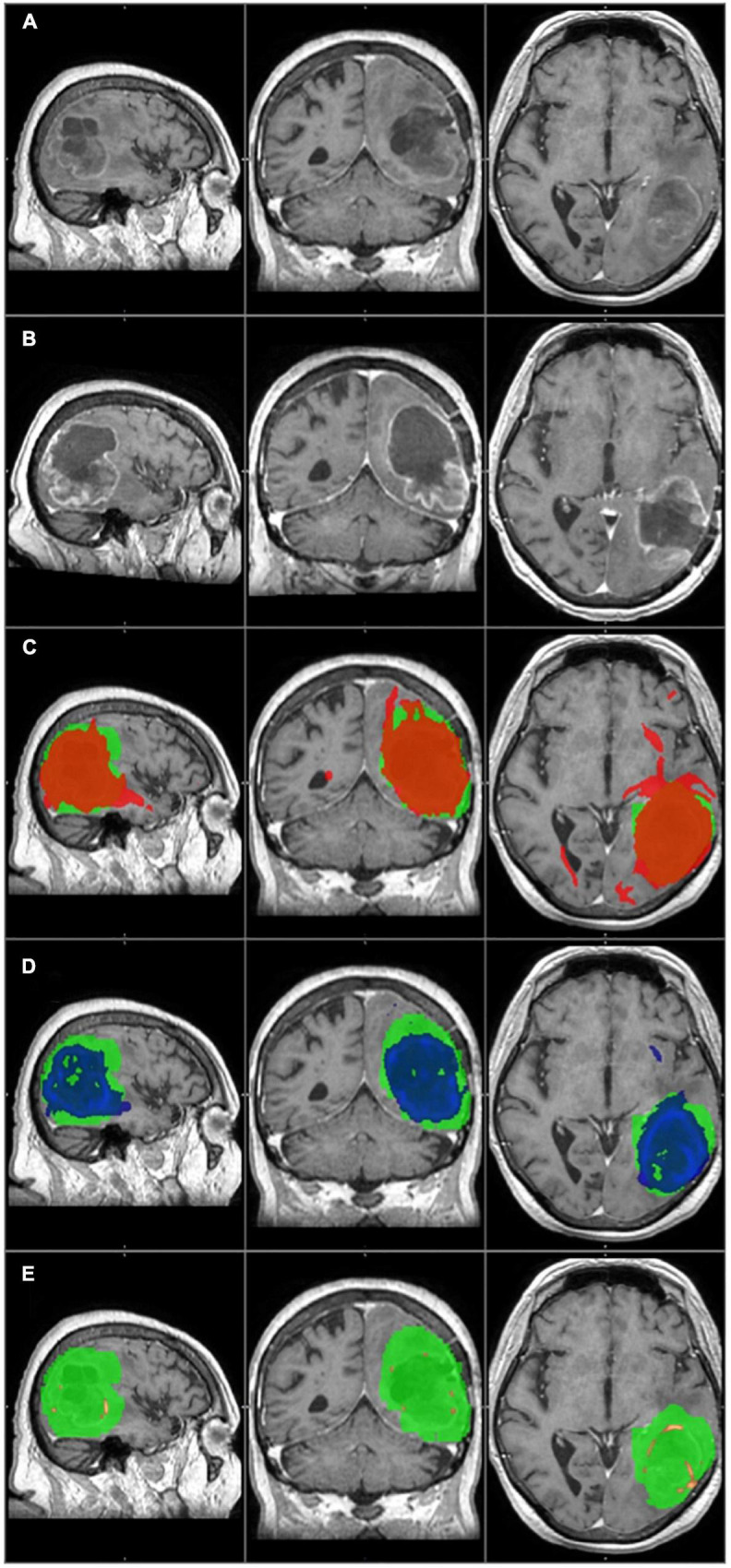
The primary tumor, the recurrence tumor, and the overlap between the different TCs and the tumor recurrence mask of the representative case of Patient 14 are displayed in this figure. All images are transformed to the MNI152 1 mm space. **(A)** The preoperative contrast enhanced T1 image. A huge tumor with ring shaped contrast enhancement can be seen in the left temporo-parito-occipital region. **(B)** The size and the location of the tumor recurrence. **(C)** The 1% TC (red) is overlapped on the tumor recurrence mask (green). **(D)** The 5% TC (blue) is overlapped on the tumor recurrence mask (green). **(E)** The 40% TC (red-yellow) is overlapped on the tumor recurrence mask (green). **(C–E)** Are on the preoperative T1 images. Although the sensitivity is higher at 1% than at 5%, the specificity is lower due to the greater number of false positive regions. The best overlap can be seen at the 5% threshold level. At 40%, the TC is almost invisible and covers only a small portion of the tumor recurrence mask resulting in high specificity but low sensitivity.

**FIGURE 3:**
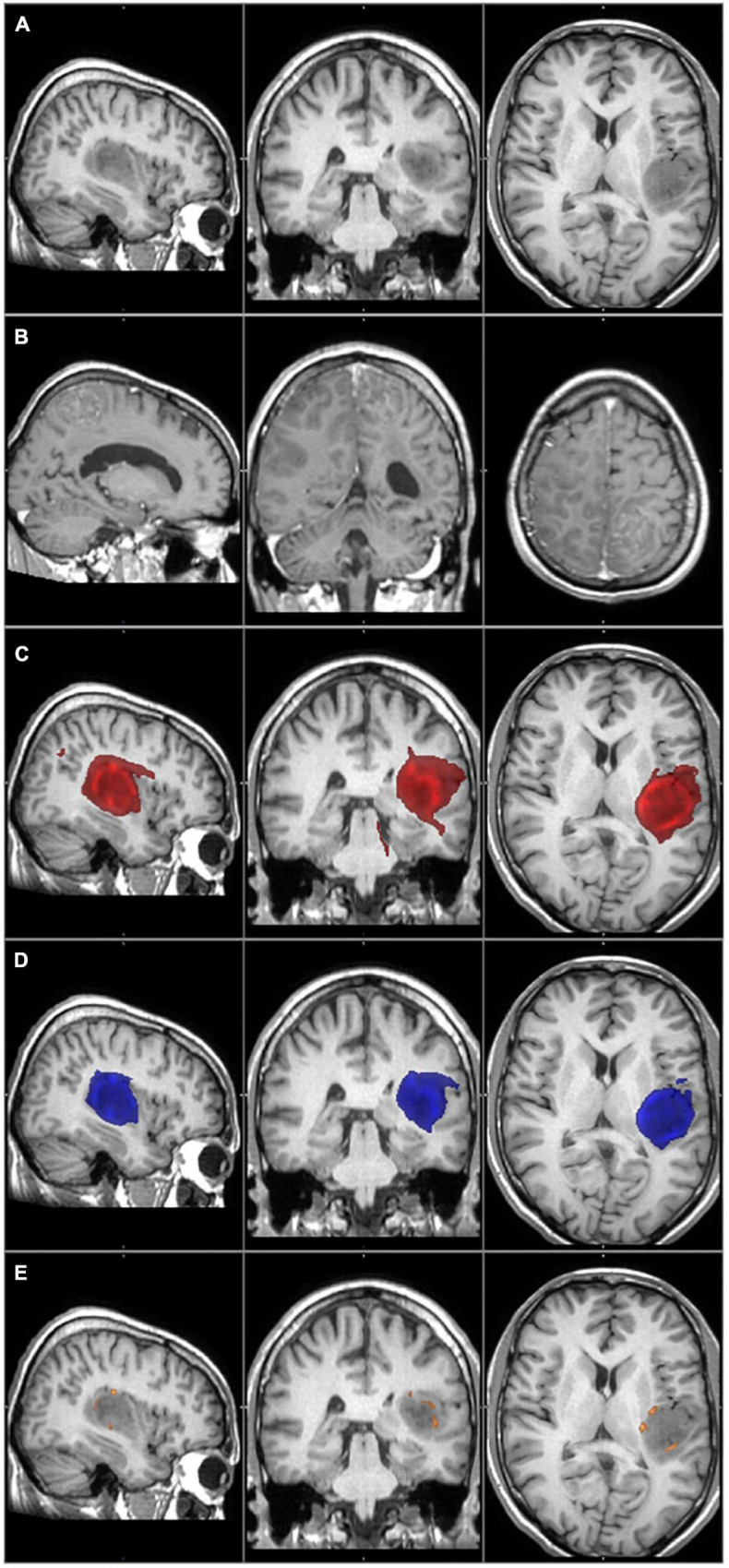
The primary tumor, the recurrence tumor, and the overlap between the different TCs and the tumor recurrence mask of the representative case of Patient 1 are displayed in this figure. All images are transformed to the MNI152 1 mm space. **(A)** The preoperative contrast enhanced T1 image. The tumor is located in the left temporo-parietal region and does not enhance the contrast agent, but histology verified the glioblastoma. **(B)** The size and the location of the tumor recurrence. **(C)** The 1% TC (red) is overlapped on the tumor recurrence mask (green). **(D)** The 5% TC (blue) is overlapped on the tumor recurrence mask (green). **(E)** The 40% TC (red-yellow) is overlapped on the tumor recurrence mask (green). **(C–E)** Are on the preoperative T1 images. The main part of the recurrence is far away from the original tumor location, which leads to very low sensitivity even at the 1% threshold level.

**FIGURE 4:**
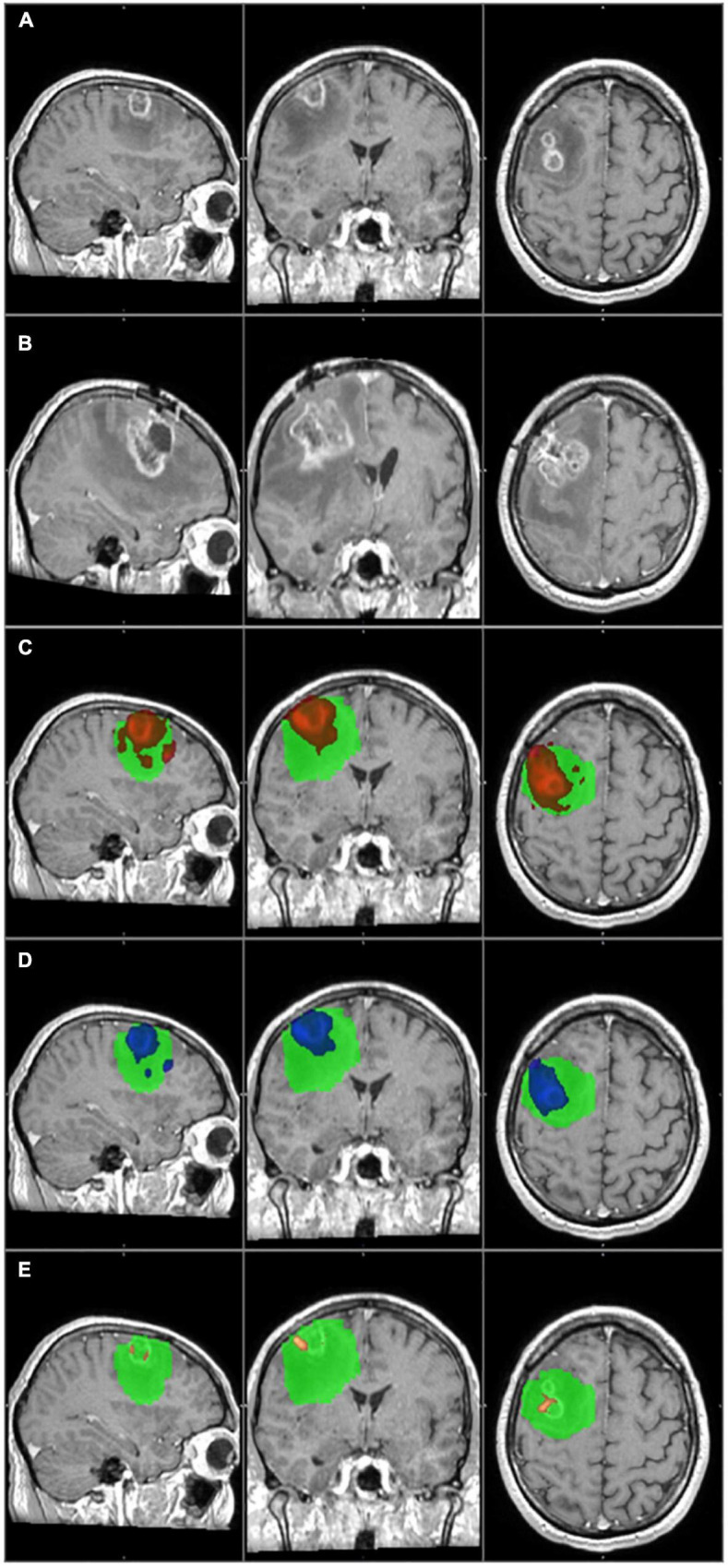
The primary tumor, the recurrence tumor, and the overlap between the different TCs and the tumor recurrence mask of the representative case of Patient 20 are displayed in this figure. All images are transformed to the MNI152 1 mm space. **(A)** The preoperative contrast enhanced T1 image. Two small tumors with ring shaped contrast enhancement can be seen in the right frontal region. **(B)** The size and the location of the tumor recurrence. **(C)** The 1% TC (red) is overlapped on the tumor recurrence mask (green). **(D)** The 5% TC (blue) is overlapped on the tumor recurrence mask (green). **(E)** The 40% TC (red-yellow) is overlapped on the tumor recurrence mask (green). **(C–E)** Are on the preoperative T1 images. As it can be seen, the TCs are fully overlapping with the tumor recurrence mask and therefore the specificity is 100% at every threshold. On the other hand the recurrence remarkably exceeds the border of the TCs, and the sensitivity is low, even at 1%. Please note that the direction of the recurrence is in correspondence with the TCs, and the low sensitivity is the result of the fast tumor progression.

The primary tumor and the tumor recurrence masks were created on the patients’ preoperative and follow-up T1 images, respectively. The contrast-enhancing part was considered to be the tumor, and it was manually delineated by two independent researchers. The primary tumor mask was used as the seed mask for tractography analyses. Figures 2–4.

### Tractography analyses

Based on a multifiber model, probabilistic tractography was performed using the primary tumor mask (Behrens et al., 2007). The default settings of the FDT (5,000 samples, 0.5-mm step length, curvature threshold = 0.2) were applied (Behrens et al., 2003a,b). It resulted in a probabilistic tract which is a set of voxels with different probabilities of connection to the original seed mask. The tractography result was called tumor connection (TC).

To eliminate low-probability connections (false positive results), threshold levels were set to include only those voxels that represented a connectivity value equal to or greater than a certain percentage of the maximum connectivity voxel of the TC (Bennett et al., 2011; Khalsa et al., 2014).

In this work, we defined connectivity as the total number of successful samples per voxel. Thirteen threshold levels were tested resulting in thirteen TCs: 1, 5, 10, 15, 20, 25, 30, 40, 50, 60, 70, 80, and 90% (Figures 2–4). We assessed which of the above threshold levels has the highest sensitivity and specificity in predicting the true extent of the tumor. The area of tumor recurrence was considered the primary infiltrated brain area, which therefore corresponded to the original true extent of the glioblastoma.

### Defining sensitivity and specificity

To validate the reliability of this method in the prediction of glioblastoma recurrence, sensitivity and specificity were defined at each threshold level in each patient. Individual and group results were also calculated.

All thirteen thresholded TCs and the tumor recurrence mask were transformed to the standard space.

The standard cortical and subcortical brain regions were projected on the patients’ brain in the MNI152 space and it was then assessed how many of them overlapped with the thirteen different TCs and the tumor recurrence mask.

Brain regions that overlapped with the TCs but not with the tumor recurrence mask were considered false positive results.

Brain regions that overlapped with the tumor recurrence mask but not with the TCs were considered false negative results.

Sensitivity and specificity were calculated using the following formula:

Sensitivity = A/(A + C)

Specificity = D/(D + B)

A: number of brain regions covered by both TC and tumor recurrence mask.

B: number of brain regions only covered by the TC.

C: number of brain regions only covered by the tumor recurrence mask.

D: number of brain regions covered by neither the TC nor the tumor recurrence mask.

The complete analysis (preprocessing steps, defining masks, tractography) took approximately 1 h for each patient. Probabilistic tractography was performed using a GPU-based implementation of BEDPOSTX and PROBTRAKX.

## Results

In our study group female-to-male ratio was 1:3. Average age was 51.5 years. A total of 50% of the patients was between 45 and 60 years old. Most frequently the tumor was located in the left hemisphere (75%) and in the frontal lobe (45%). The two most common neurological deficit was hemiparesis (35%) and speech disturbancies (25%). A total of 40% of the patients did not have severe neurological symptoms preoperatively. The average KPS was 73% preoperatively and 80% at 2 months after the operation. Navigation guided (Medtronic Inc StealthStation iNav or S8) individually planned minimal invasive craniotomy was performed in all cases. Total (70%) or subtotal (30%) resection was achieved in all patients. Four patients (20%) had grade III anaplastic oligodendroglioma (Table 1). The progression-free and overall survival periods, age, and female-to-male ratio corresponded to the literature (Ostrom and Barnholtz-Sloan, 2011; Michaelsen et al., 2018).

There were two outliers in the study group (Patients 1 and 20) who showed remarkably lower sensitivity than the rest of the patients (Table 2 and Figures 3, 4). Their results were excluded from the group analyses as it is explained in the section “Discussion.”

**TABLE 2:** The sensitivity and specificity values are listed in this table at each threshold level of the three example cases.

	1%	5%	10%	15%	20%	25%	30%	40%	50%	60%	70%	80%	90%
**A**													
	0,94	0,80	0,77	0,74	0,66	0,63	0,57	0,46	0,23	0,20	0,14	0,11	0,09
Specificity	0,85	0,96	0,98	0,99	1,00	1,00	1,00	1,00	1,00	1,00	1,00	1,00	1,0
**B**													
Sensitivity	0,36	0,27	0,23	0,23	0,23	0,23	0,18	0,18	0,09	0,09	0,09	0,05	0
Specificity	0,79	0,85	0,89	0,90	0,90	0,90	0,91	0,91	0,92	0,94	0,96	0,99	0,99
**C**													
Sensitivity	0,44	0,25	0,25	0,19	0,19	0,19	0,19	0,19	0,19	0,19	0,13	0,13	0
Specificity	1	1	1	1	1	1	1	1	1	1	1	1	1

A: The results of Patient 14 are in concordance with the group average sensitivity and specificity values. B: The sensitivity and specificity of Patient 1. Please note that the specificity is high but the sensitivity is low even at 1% threshold level. This is because there is a smaller part of the recurrent tumor at the original site, which is covered by the TCs, therefore the ratio of the false positive results is low. The main part of the recurrence is far away from the original site, and this will result in a high false negative result. C: The results of Patient 20 are summarized in this table. The specificity is 100% at all threshold level while the sensitivity is very low even at 1%. This is due to the extensive local tumor recurrence which overlaps totally with all TCs. It also exceeds its borders and affects several other brain regions resulting in a high rate of false negative results.

A total of 18 patients’ data were averaged and evaluated. TCs with higher threshold levels were associated with higher specificity and lower sensitivity. The maximum sensitivity and specificity were observed at 1 and 90%, respectively (Table 3).

**TABLE 3:** The group average sensitivity and specificity values (with standard error of the mean) are listed in this table at each threshold level.

	1%	5%	10%	15%	20%	25%	30%	40%	50%	60%	70%	80%	90%
**Sensitivity (Avg ± SEM)**	0,91 ± 0,03	0,81 ± 0,04	0,73 ± 0,04	0,67 ± 0,05	0,61 ± 0,05	0,60 ± 0,05	0,58 ± 0,06	0,50 ± 0,06	0,35 ± 0,05	0,31 ± 0,06	0,21 ± 0,06	0,14 ± 0,05	0,13 ± 0,06
**Specificity (Avg ± SEM)**	0,81 ± 0,03	0,9 ± 0,02	0,93 ± 0,02	0,95 ± 0,01	0,96 ± 0,01	0,96 ± 0,02	0,92 ± 0,05	0,93 ± 0,05	0,98 ± 0,01	0,94 ± 0,05	0,94 ± 0,05	0,94 ± 0,05	0,94 ± 0,05

As the threshold increases the sensitivity decreases and the specificity increases. The highest values can be seen at 1 and 5% threshold levels.

**GRAPH 1:**
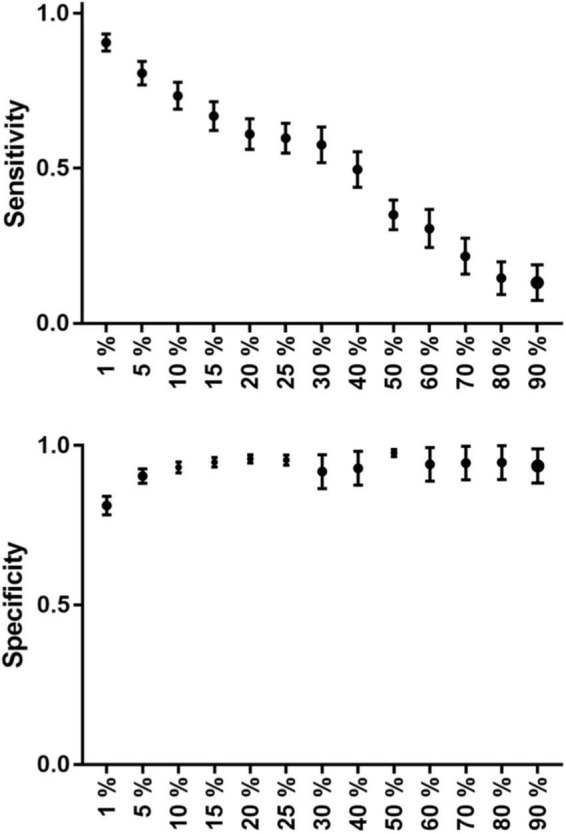
The group average sensitivity and specificity values (with standard error of mean) can be seen on this graph at each threshold level. Sensitivity decreases in a nearly linear fashion, while specificity has a nearly parabolic increase along the increasing threshold levels.

The TC with the 5% threshold level seems to give the most reliable results. In the majority of the patients (72.2%), sensitivity was higher than 75%. Additionally, in 77.7% of patients, specificity was greater than 85%. At the group level, both measures were higher than 80%. Average sensitivity was 81% and average specificity was 90% at the 5% threshold (Table 3 and Graph 1 and Figure 2).

Tumor volume varied between 6.2 and 116.4 cm^3^. The volume of the recurrent tumor also showed high variability, as it ranged from 7.8 to 140.2 mm^3^.

## Discussion

Glioblastoma is often considered the most aggressive type of cancer. Overall survival is 18 months and only 1% of the patients are alive 5 years after the diagnosis.

The gold standard therapy for glioblastoma is maximum safe resection followed by oncological and oncoradiological treatment. There is a positive correlation between the extent of primary tumor resection and the overall survival (Wykes et al., 2021).

In oncological surgery, an extended safety margin is used to remove the primary tumor and all the infiltrated surrounding tissues. Unfortunately, this approach cannot be employed in the case of primary brain tumors. Resection should be limited to the tumor mass-normal tissue border (Yasargil, 1994). The removal of healthy brain tissue is likely to cause severe neurological symptoms and a reduced quality of life. The remaining tumor cells infiltrating the normal brain tissue should be treated with oncological procedures, such as irradiation and chemotherapy (Stupp et al., 2005).

Glioblastoma, which is the most aggressive type of these infiltrative primary brain tumors, recurs from the remaining infiltrative tumor cells (Petrecca et al., 2013). Currently, the radiological modality with the highest sensitivity for glioblastoma is MRI (Shukla et al., 2017). The tumor mass is visible on the contrast-enhanced T1 images, but the cells infiltrating the otherwise normal brain tissue (which can be at a distance of several centimeters from the contrast enhancing border) are not.

Theoretically, if the true extent of the glioblastoma can be revealed, prolonged progression-free and overall survival periods can be achieved with supratotal resection and individually planned radiation therapy.

In the recent decade, FLAIR, MRS and amino acid-based PET-CT were introduced to identify the true extent of glioblastoma as potential imaging modalities.

In case of glioblastoma the hyperintense volume in the FLAIR images corresponds to microscopic tumor infiltration and edema, and usually exceeds the contrast enhancing volume (Watanabe et al., 1992). Preoperative FLAIR images can be used to plan surgical resection and irradiation therapy but there is no clear evidence that it has a positive prognostic value (Garrett et al., 2017; Altieri et al., 2019). The extent of FLAIR signal hyperintensity prior to the initial surgery is not associated with survival. On the other hand, overall survival is significantly affected by the preoperative contrast enhancement volume (Altieri et al., 2019). In addition, FLAIR images can identify tumor progression earlier than contrast enhanced T1 on the follow-up MRI and associated with progression free and overall survival (Garrett et al., 2017). It is hard or even impossible to differentiate where microscopic tumor infiltration ends within the edema. Identifying the exact borders of the infiltrative part of the glioblastoma is challenging on FLAIR. Therefore the true extent of the tumor can be overestimated due to false positive hyperintense regions. Probabilistic tractography has the advantage that its result can be thresholded, and not like on FLAIR, the false positive results can be ruled out.

Magnetic resonance spectroscopy has promising results to predict the site of GBM recurrence. In one study LNR MRS has 88.8% sensitivity and 97.6% specificity to detect tumor vs. normal brain tissue and 71% true and 10% false positive ratio to predict the site of glioblastoma recurrence (Deviers et al., 2014). The spatial resolution of MRS was very low (6.25 mm × 6.25 mm × 25.0 mm = 976,5 mm^3^) in this study (Ken et al., 2013). These factors make it challenging to use MRS results in routine patient care and image guided surgical and radiation therapy. Diffusion MRI based tractography on the other hand has much better resolution, which makes it a more feasible method.

^11^C- Methionin-, ^18^F-DOPA, and ^18^F-FET-PET have been introduced for primary and secondary brain tumor detection. The increased uptake in brain tumors is based on the overexpression of the amino acid transporter in the tumor cells and in the tumor supplying vessels, which is independent from the blood-brain barrier (BBB). Therefore, these amino-acid based PET methods are appropriate for the following goals: to help differentiate necrosis and tumors, provide information on changed biochemical processes prior to the appearance of morphological changes, and to characterize the residual brain tumor volume for more accurate post-operative radiotherapy.

^18^F-FET-PET has been used in some studies to determine the tumor and/or residual tumor volume for radiotherapy. In most cases, the target volume for irradiation determined this way was larger than the one determined with MRI alone (Munck Af Rosenschold et al., 2015). Post-operative ^18^F-FET-PET scans had a higher sensitivity for detecting residual tumor than MRI (Buchmann et al., 2016). Nonetheless, ^18^F-FET-PET had a sensitivity of 87% and a specificity of 68% for detecting glial tumors (Hutterer et al., 2013). These results make ^18^F-FET-PET a potential diagnostic tool to identify the true extent of glioblastomas.

There are several disadvantages of ^18^F-FET-PET that make its routine clinical use difficult. It is quite expensive, time-consuming (the total examination time can be several hours), and exposes the patient to radiation. Also, spatial resolution is limited to 5 mm. MRI, on the other hand, uses no radiation, has high spatial resolution (<1 mm), and takes about 15–20 min. It is also a part of the standard diagnostic protocol for glioblastoma and therefore does not require any extra procedures. If an MRI modality that is suitable for identifying the true extent of glioblastomas could be found, it would be a much more feasible examination to be used in clinical practice than ^18^F-FET-PET (Muoio et al., 2018).

Tractography is an advanced MRI imaging modality. It is based on diffusion MRI images. The direction of the diffusion movement of water molecules in the brain can be reconstructed. In the white matter, this movement tends to be parallel to the fiber tract orientation. Using this information, white matter pathways can be reconstructed mathematically. The major drawback of tractography is that the diffusion direction can be uncertain in several important brain regions (e.g., white and gray matter border, gray matter, basal ganglia, crossing fiber tracts, tumor, peritumoral edema) and, therefore, the result is unreliable (Bammer, 2003; Wakana et al., 2004; Johansen-Berg and Behrens, 2006). Probabilistic tractography is one possible algorithm to overcome this limitation. It takes the uncertainty of water diffusion into account. The result of probabilistic tractography is a set of voxels. Each voxel has a value representing the probability that the given voxel belongs to the visualized white matter fiber tract. This basically means that all voxels can be identified, even the ones that have the lowest chance of being connected to the tract. It also means that there are a lot of false positive results. Therefore, an optimal threshold level must be defined to exclude the highest number of false positive and false negative voxels.

Glioblastoma originates from a core. As it progresses, a tumor mass and an infiltrative part of the tumor develop (Wick et al., 2018). Due to the fast-growing nature of the tumor, the center of the mass is often necrotized, and the edges consist of viable tumor (Young, 2007). The infiltrative part is a mixture of tumor cells and white matter fiber tracts. The two parts form a structural unit and can be considered an abnormal fiber tract that has several connections with the surrounding pathways (Osswald et al., 2015). This makes probabilistic tractography a potential tool to identify the connections between the tumor and its pathways, furthermore, the infiltrated brain regions and, more importantly, the true extent of the tumor could be pinpointed (Mukherjee et al., 2008).

The problem is that when probabilistic tractography is performed, the infiltrative tumor part is invisible on standard MRI images (Lasocki and Gaillard, 2019).

When the remaining tumor cells become resistant to the oncological treatment, they start to proliferate (Milano et al., 2010; Petrecca et al., 2013). The infiltrative part increases in volume and then becomes visible on conventional MRI images. Intuitively, the tumor becomes visible in the brain regions where it was invisible on the initial and previous follow-up images. The identified tumor recurrence then can be used for comparison with the TCs identified using probabilistic tractography.

We had to find a method that would allow us to reliably compare the TCs with the tumor recurrence mask. It would be a simple way to define the overlapping volume of the two and then, based on the ratio of the overlap, calculate sensitivity and specificity. Unfortunately, this method would not give us a reliable result because of the following two limiting factors. First, in most of the cases, the anatomy of the brain gradually changes during the time between the preoperative and the follow-up MRI scan. It is challenging, even with non-linear registration, to create perfect alignment between the preoperative and follow-up MRI images because of the mass effect of the tumor. Therefore, a direct comparison of the anatomical location of the TCs and the tumor recurrence mask is unreliable. The second limitation is due to the nature of tractography and the natural course of glioblastomas. TC volumes change by thresholding. The recurrence volume depends on when the follow-up MRI scan was performed. Contrast enhancement first appears along the fiber tracts, then the tumor gets thicker and soon exceeds the volume of the fiber tract. Accordingly, infiltrated fiber tracts may be visible on the tractography images, but the volume may differ remarkably from the tumor recurrence mask.

Therefore, instead of using the volumes for comparison, we identified brain regions affected by both the tumor and the TCs. According to the JHU ICBM–DTI–81 white matter label and AAL3 standard atlases, the standard brain was divided into subcortical and cortical regions, and the number of affected regions was calculated.

At the group level, we found high sensitivity (81%) and specificity (90%) rates at the 5% threshold level. Our results suggest that the presented method is a reliable and clinically feasible way to predict the true extent of glioblastomas.

On the other hand, there are several limiting factors of this method that can affect the final result.

As it has been previously mentioned, the preoperative and follow-up MRI images do not align perfectly with the standard MRI images. This means that the TCs and the tumor recurrence mask are not completely in an anatomically identical location. This problem cannot be completely eliminated by neither of the widely available registration algorithms. The misalignment can only be minimized to have the least effect on the results (Kocher et al., 2021).

Sensitivity and specificity are directly determined by the volume and extension of the tumor recurrence mask, which depend on the time of the follow-up MRI scan. In case of a later MRI scan, the tumor volume can be significantly higher; consequently the calculated sensitivity and specificity can also be different.

To overcome these two limitations, we did not make a direct comparison between the volumes of the TCs and the tumor recurrence mask but counted the number of affected cortical and subcortical areas within the standard space. Registering the preoperative and follow-up MRI images to the standard space reduces the anatomical distortion between the two to the possible minimum. Even if the TCs and the tumor recurrence mask are in an anatomically identical location, the volume difference between the two can change the results. Therefore, we did not compare the volumes but the number of the affected brain areas instead. This way, if either the TC or the tumor recurrence mask reached one area, it was counted as positive. The difference in the volumes reaching the same location did not matter. Only the positive hit counts. Unfortunately, even this technique cannot perfectly rule out the above mentioned limitations. A good example for this is the case of Patient 20 (see below).

There is another limitation to the presented method, which is related to the nature of the tumor recurrence. The two outlier patients are perfect examples to that. The appearance of the recurrence on the follow-up MRI was not eligible to determine which part of the brain was infiltrated preoperatively. In their case, the presented method was unreliable to define the specificity and sensitivity. Their data were excluded from the group average to avoid false distortion of the results.

Patient 1 had a rare type of tumor progression. The recurrence appeared in a multifocal fashion, far away from the original site (Figure 3).

Patient 20 had a recurrence in the white matter around the resection cavity. The contrast enhancing part of the original tumor was very small (6.2 cm^3^) if compared to the recurrence volume (54.9 cm^3^). A huge area of the frontal lobe was included by the tumor recurrence mask but not by TCs (Figure 4), and there was a considerable difference in the number of affected brain regions.

In both cases, the special anatomical situations mentioned above resulted in a low level of overlap between the TCs and the tumor recurrence masks, therefore they decreased the sensitivities. In the case of Patient 1, the distant infiltrated areas could not be identified based on the tractography images, and the low sensitivity value was real. However, in the case of Patient 20, it was a partially false result. At the time of the follow-up MRI tumor progression was significant. The TCs overlapped with several brain regions where the recurrence appeared, and the direction of the recurrence was in concordance with the TCs. On the other hand, the tumor recurrence also contained many brain regions away from the original site. This resulted in a high number of false negative results and low sensitivity, even though tractography predicted the location of the recurrence well. The case of Patient 20 enlightens the importance of the timing of the follow-up MRI in our methodology. An earlier follow-up MRI with a less prominent tumor progression may have resulted in a better correlation with the TCs. In conclusion we believe that in the case of Patient 20, the TC with the 5% threshold level would give us reliable information regarding the true extent of the tumor, but because of the reason discussed above, the calculated sensitivity is low (Table 2 and Figure 4).

On the other hand, the two outlier patients also highlight the limitation of the applicability of the presented method. It provides useful information in only those glioblastoma patients who have typical progression pattern and the recurrence appears within the close proximity of the resection cavity. According to the literature 90–95% of the patients belongs to that group (De Bonis et al., 2013). Our study group is in concordance with that, as 90% of the patients had the recurrence around the resection cavity.

In our study, we presented a probabilistic tractography-based method to identify the true extent of glioblastomas at the time of diagnosis. With the presented method, we were able to visualize the infiltrated brain areas on the preoperative MRI images in most patients with high sensitivity and specificity rates. It may help achieve more radical tumor resection and individually planned radiation therapy, which may lead to prolonged survival. As the presented work was a pilot study to validate the methods’ reliability in terms of identification of the true extent of the glioblastoma, a prospective study is planned to investigate the potential positive effect of its application on the survival of glioblastoma patients.

## Data availability statement

The original contributions presented in this study are included in the article/supplementary material, further inquiries can be directed to the corresponding author.

## Ethics statement

The studies involving human participants were reviewed and approved by the Local Ethical Review Board of the University of Szeged, Szeged, Hungary. The patients/participants provided their written informed consent to participate in this study.

## Author contributions

DK contributed to conception, design and study, data analyses, and wrote the first version of the manuscript. LS, BS, and MR organized the database. LS performed statistical analyses and created figures and tables. KH and AM collected patients’ data and created tables. PB organized the study. All authors contributed to the article and approved the submitted version.

## Footnotes

1
www.fmrib.ox.ac.uk/fsl

## Abbreviations

AAL3: automated anatomical labeling atlas 3
ASSET: array coil spatial sensitivity encoding
BEDPOSTX: bayesian estimation of diffusion parameters obtained using sampling techniques
DICOM: digital imaging and communications in medicine
DTI: diffusion tensor imaging
DWI: diffusion weighted images
F-DOPA: fluoro-dihydroxyphenylalanine
F-FET: F-Fluoro-Ethyl-Tyrosine
FDT: FMRIB’s diffusion toolbox
FLAIR: fluid attenuation inversion recovery
FMRIB: Functional magnetic resonance imaging of the brain
FOV: field of view
FSL: FMRIB software library
FSPGR: fast spoiled gradient echo
GE: general electric
GPU: graphical processing unit
IDH: isocitrate dehydrogenase mutation
JHU-ICMB-DTI: Johns Hopkins University, international consortium of brain mapping, diffusion tensor imaging
LNR: lactate/N-acetylaspartate ratio
MGMT: O^6^-methylguanine-DNA methyltransferase
MNI152: Montreal neurological institute 152
MRI: magnetic resonance imaging
MRS: magnetic resonance spectroscopy
NIFTI: neuroimaging informatics technology initiative
OS: overall survival
PET-CT: positron emission tomography, computed tomography
PFS: progression free survival
PROBTRACX: probabilistic tracking with crossing fibers
SWI: susceptibility weighted images
TC: tumor connection
TE: time of echo
TR: time of repetition.
